# Supplementary material for: The interactome of intact mitochondria by cross-linking mass spectrometry provides evidence for coexisting respiratory supercomplexes
Source: Mol Cell Proteomics. 2017 Dec 8;17(2):216–32. doi: 10.1074/mcp.RA117.000470 (PMC5795388; doi:10.1074/mcp.RA117.000470)
Supplement: Supplemental Data [file supp_RA117.000470_133922_0_supp_23978_fzffwf.zip › spectra_annotation/mito_spectra_annotation/123-1-1-1-10-1.pdf]

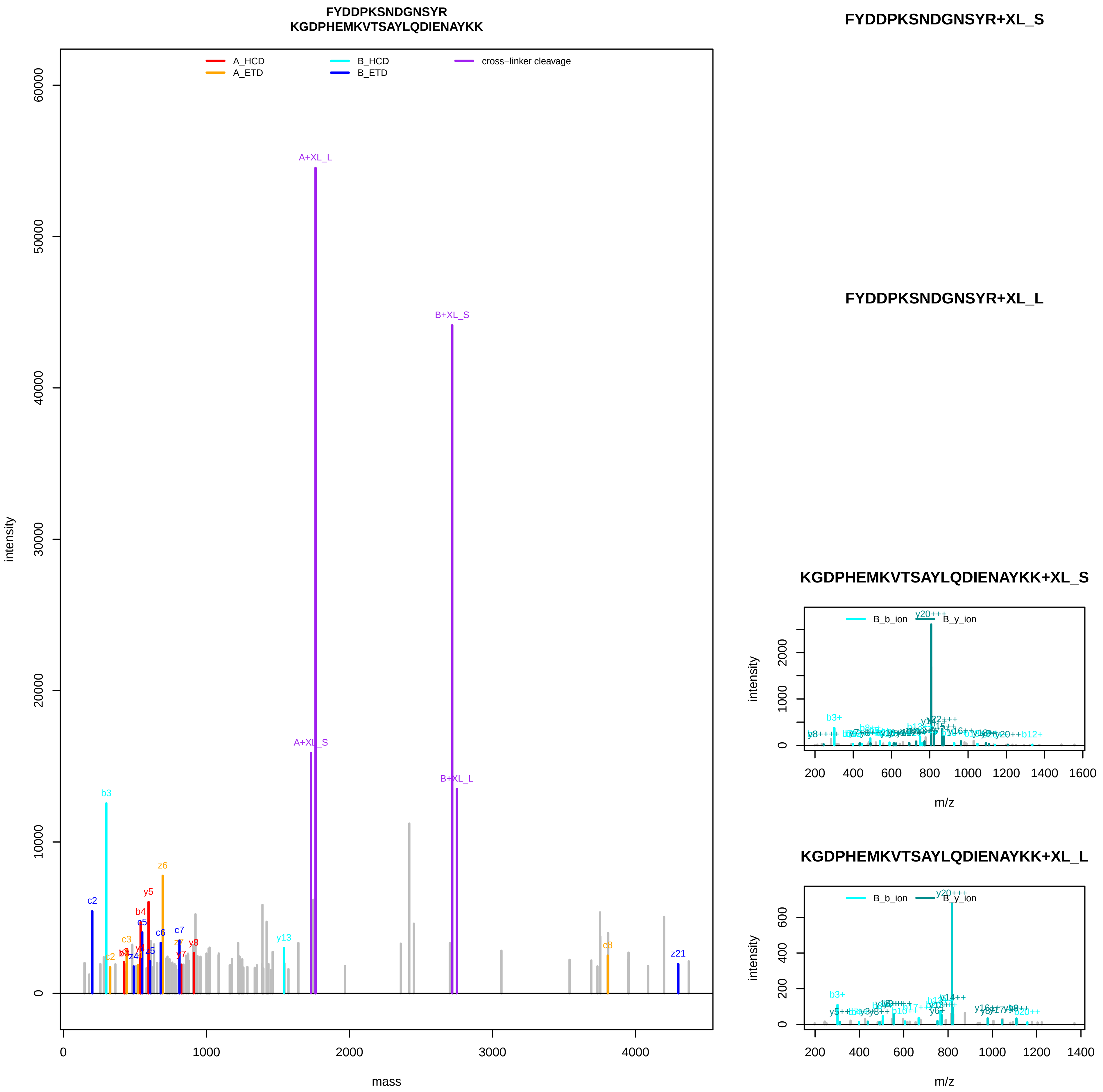
FYDDPKSNDGNSYR
KGDPHEMKVTSAYLQDIENAYKK
A_HCD
A_ETD
B_HCD
B_ETD
cross−linker cleavage
A+XL_L
B+XL_S
A+XL_S
B+XL_L
b3
c2
z6
y5
b4
c5
c3
c6
c7
y8
y7
y13
c8
z21
intensity
mass
0
1000
2000
3000
4000
10000
20000
30000
40000
50000
60000
FYDDPKSNDGNSYR+XL_S
FYDDPKSNDGNSYR+XL_L
KGDPHEMKVTSAYLQDIENAYKK+XL_S
B_b_ion
B_y_ion
y20+++
b3+
b12+
m/z
200
400
600
800
1000
1200
1400
1600
2000
KGDPHEMKVTSAYLQDIENAYKK+XL_L
y14++
y6+
b20++
